# In vitro regeneration and *Agrobacterium*-mediated genetic transformation of Dragon’s Head plant (*Lallemantia iberica*)

**DOI:** 10.1038/s41598-022-05776-w

**Published:** 2022-02-02

**Authors:** Rahman Ebrahimzadegan, Asad Maroufi

**Affiliations:** 1 Department of Plant Production and Genetics, Faculty of Agriculture, University of Kurdistan, Sanandaj, Iran; 2 Research Center for Medicinal Plant Breeding and Development, University of Kurdistan, Sanandaj, Iran

**Keywords:** Plant biotechnology, Plant breeding, Plant cell biology, Plant molecular biology

## Abstract

Dragon’s head plant (*Lallemantia iberica*), is a flowering species belongs to the mint family (Lamiaceae). The species contains valuable essential oils, mucilage and oil which are used in pharmaceutical and food industries. Tissue culture is a feasible strategy to attain large‐scale production of plantlets with a huge potential to produce plants with superior quality. The objective of this study was to develop a simple and efficient method for regeneration and transformation of *L. iberica*. To reach this goal, the regeneration ability of various explants including leaf, cotyledonary node, hypocotyl and cotyledon segments was investigated in MS medium supplemented with diverse concentrations of NAA (Naphthalene acetic acid) and BAP (6-Benzyl Amino Purine). According to the results, cotyledonary nodes showed the best regeneration response. The maximum rate of regeneration (and number of induced shoots was achieved in 1 mg l^−1^ BAP in combination with 0.05 mg l^−1^ NAA from the cotyledonary nodes. Additionally, through the optimized regeneration technique *Agrobacterium*-mediated transformation of *L. iberica* was successfully accomplished. Gene transfer was assessed on leaf samples from regenerated plantlets under a fluorescent microscope to detect the GFP signals. Moreover, transgene integration and its expression were confirmed by PCR and RT-PCR analysis, respectively. The establishment of these efficient regeneration and genetic transformation methods paved the way for further application such as plant improvement, functional analysis and gene editing.

## Introduction

*Lallemantia iberica*, known generally as dragon's head, is a herb belonging to the mint family (Lamiaceae), originated from Caucasus and Middle East regions and widely distributed in Europe and some western areas of Asia^1^. *L. iberica* seeds are used in traditional medicine as stimulant, reconstitute, expectorant and diuretic. Its seeds contain large fatty acids like palmitic 6.5%, stearic 1.8%, oleic 10.3%, linoleic 10.8% and linolenic 68%^2^. Additionally Dragon’s head plant comprises valuable secondary metabolites such as p-cymene, isophytol, t-cadinol, 3-octanol, terpinen-4-ol^3^. Moreover, its seed mucilage is used in the treatment of nervous, hepatica and renal diseases^4^. Additionally, this herb is mainly harvested for its seeds since they contain valuable oil compounds around 30% drying weight holding high amount of valuable omega 3 fatty acid and alpha-linolenic acid (67–74%) which has many applications in pharma and food industries^4,5^. *Lallemantia iberica* is an attractive plant and because of its ornamental value it is used for landscaping and urban horticulture at various places^6^.

Since there is growing concerns over the side effects of chemical medications and the cost of these drugs, more attention has been focused on the use of natural and plant-derived compounds as an alternative or supplement. Therefore, the willingness to use medicinal plants and plant-derived secondary metabolites is increasing in the world. To meet this demand, cultivation of more medicinal plants or tissue culture to produce more natural products is essential. Plant tissue culture technique has several advantages and therefore, it has been used as a main platform for secondary metabolites production. In addition, plant tissue culture is considered to be the most efficient technology which used for basic and applied purposes such as, study of plant developmental processes, functional gene studies, micropropagation, generation of transgenic plants, plant breeding, virus-free plants production, preservation and conservation of germplasm of plants, and rescue of threatened or endangered plant species^7,8^. Establishment of a reliable in vitro plant regeneration protocol is usually a prerequisite to the genetic transformation^9^. Improvement of plants with potential desired traits for instance greater quality, high yield, enhanced adaptability, disease and stress resistance based on plant biotechnological tools such as transformation is mostly depended on tissue culture technique^10–12^. Genetic transformation enables the introduction of foreign genes into *L. iberica* or efficient modification of target genes to create new cultivar with desirable traits such as higher valuable secondary metabolites or omega 3 fatty acid and alpha-linolenic acid. Moreover, in vitro regeneration and genetic transformation are important tools to enhance the production of secondary metabolites and engineer the valuable oil content in plants such as *L. iberica*. Actually *Agrobacterium*-mediated transformation has been frequently used to improve many plant species for several valuable traits^13^. Due to significant advantages of transformation using *Agrobacterium*, such as integration of transgene in the genome with low copy number, transgene stability in the host genome and efficient transformation, this is a preferred method comparing with other transformation systems^14,15^.

To the best of our knowledge, no well-organized method for transformation of *L. iberica* medicinal plant via *Agrobacterium tumefaciens* is available and only very limited studies have been performed on its regeneration^6,16^ and so far,. Therefore, for the first time an appropriate technique for in vitro regeneration and gene delivery using *Agrobacterium* are presented. This allows genetic improvements to be put into practice for *Lallemantia iberica*. In summary, the major factors which affect the regeneration and transformation such as, type of explants, concentrations or combinations of PGRs, acetosyringone, *Agrobacterium* inoculation duration, have been successfully optimized and a viable regeneration and transformation protocol well established.

## Material and methods

### In vitro seed germination

Dragon’s Head plant seeds were procured from Pakanbazr an Iranian private joint stock company, providing deferent kinds of seeds to the customers (www.pakanbazr.com/en/lallemantia-iberica-seed). Using these seeds for research are not restricted according the role of University of Kurdistan, Sanandaj, Iran. This study meets national and international guidelines for research. The seeds were surface-sterilized by immersion in 70% ethanol for 30s and immediately washing three times with sterilized distilled water, followed by soaking in 5% sodium hypochlorite for 15 min and rinsing three times with sterilized distilled water. Next, the seeds were transferred into half strength Murashige and Skoog (MS) basal medium^17^ and incubated for germination under a photoperiod of 8/16 h dark/light using a 2400 lx light regime at 25 ± 1 °C.

### In vitro plant regeneration

21-day old seedlings grown in vitro, were used as source of explants for regeneration potential of *L. iberica* plants. The cotyledonary node, cotyledon, hypocotyl and leaf pieces were prepared in small segments about 0.5–1 cm^2^. The explants were separately cultured on MS medium containing four combinations of 6-Benzylaminopurine (BAP; 0, 0.5, 1 and 2 mg l^−1^) and 1-naphthaleneacetic acid (NAA; 0, 0.05, 0.1 and 0.2 mg l^−1^). Afterwards, all cultures were incubated under a photoperiod of 8/16 h dark/light using a 2400 lx light regime at 25 ± 1 °C. For all plant growth regulator (PGR) combinations, 10 explants were incubated in each glass vessels. The explants were sub-cultured every two weeks and the number of regenerated shoots were finally recorded.

### Root induction

The well regenerated shoots were cultured on MS supplemented with 4 concentrations of NAA (0, 0.1, 0.5 and 1 mg/l) and incubated in culture room at 25 ± 1 °C. To evaluate the optimal concentrations of NAA for root induction all samples were daily monitored and data of rooting were collected.

### Acclimatization of plantlets

The completely rooted plantlets were washed to remove the remains of MS medium, then transferred to small plastic pots containing 1:1 ratio of coco peat and perlite mix and totally covered with a transparent plastic shield. To adapt them to environmental condition, 2–3 small holes in the plastic wrap were daily created. Meanwhile, the plants were regularly irrigated with diluted liquid MS basal medium. After 3–4 weeks, the shield was entirely removed. Finally, the plantlets were transferred into the larger pots filled with 1: 1: 1 ratio of farm soil, compost and sand for more growth and evaluation.

### Agrobacterium strain and binary vector

**Figure 1:**
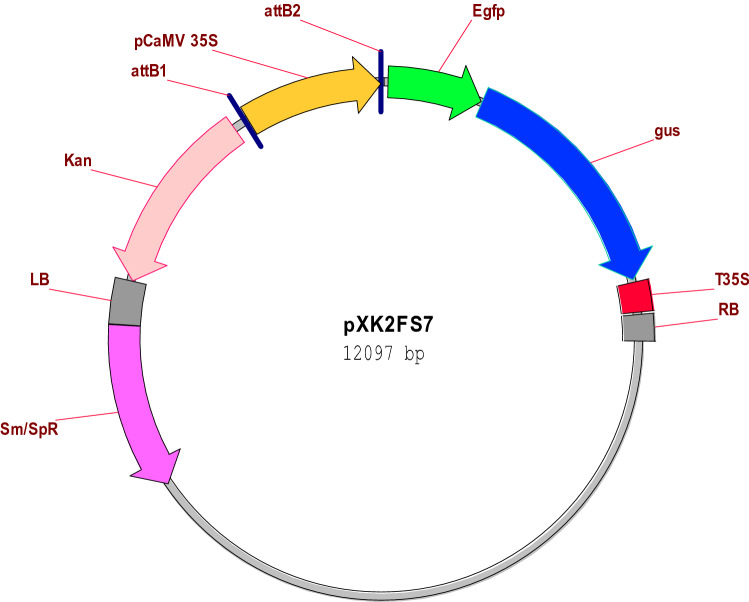
Genetic map of pXK2FS7 binary vector containing two reporter genes of GFP and GUS driven by the CaMV 35S promoter.

*Agrobacterium tumefaciens* strain C58C1Rif^R^ (pMP90) containing pXK2FS7 binary vector has been utilized for transformation. It was kindly provided by Department of Plant Systems Biology (VIB, Ghent, Belgium). The genetic map of pXK2FS7 plasmid is shown in Fig. 1. It carries two reporter genes green fluorescent protein (GFP) and β-glucuronidase (GUS) that both are controlled under the CaMV 35S promoter^18^. To prepare the suspension culture of the *Agrobacterium*, a single colony obtained from the streaked LB agar petri plate incubated at 28 °C, was first inoculated in liquid LB selective medium containing 50 mg l^−1^ rifampicin, 50 mg l^−1^ streptomycin and 25 mg l^−1^ gentamicin. Next the culture was incubated at 28 °C on a shaker with 200 rpm to achieve desired OD600 = 0.5–0.8. Later on, the cells were collected by centrifugation at 5000 rpm for 10 min. The pellet was finally re-suspended in appropriate volume of liquid MS medium (pH 7.8) for further use.

### Agrobacterium-mediated transformation of explants

The cotyledonary node pieces were submerged in *A. tumefaciens* suspension (OD_600_ = 0.6) for inoculation. The inoculated explants blotted dry on sterile Whatman paper under sterile conditions, then immediately transferred to co-cultivation medium (MS containing no PGRs and different concentrations of acetosyringone). The cultures were incubated in a dark chamber (25 ± 1 °C) for 3 days. Subsequently, the infected explants were washed three times with sterile distilled water, following with 50 ml of cefotaxime solution 500 mg l^−1^ for 20 min to remove any trace of bacteria. The explants were gently blotted dry on sterile Whatman paper and directly cultured on regeneration medium (MS containing 1 mg l^−1^ BAP and 0.05 mg l^−1^ NAA) having kanamycin at concentration of 60 mg l^−1^. After regeneration and analysis of regenerated shoots by PCR, transformation efficiency (%) was calculated as (The number of positive transgenic plants / the number of infected explants) × 100.

### Optimal kanamycin concentration

A sensitivity assessment was performed in order to find the optimal concentration of kanamycin, since it varies from species to species. The cotyledonary node explants were primarily cultured on regeneration medium containing different concentrations of kanamycin (0, 30, 60, 90 and 120 mg l^−1^) and the number of survived explants was finally recorded for each kanamycin concentration.

### Effect of infection time on transformation

To find the optimal infection time for transformation of *L. iberica*, in a separate experiment, explants were immersed in infective suspension of *Agrobacterium* (OD _600_ = 0.6) without acetosyringone for 4 certain infection time 10, 15, 20 and min. Following the transformation of explants, transformation efficiency was calculated.

### Effect of acetosyringone on transformation

To study the effect of acetosyringone on transformation frequency, in a separate experiment, the suspension of *Agrobacterium* cells was supplemented with different concentration (0, 100, 200 and 300 µM) of acetosyringone for 15 min. Following the transformation of explants, transformation efficiency was calculated.

### Green fluorescent protein visualization

Green fluorescent protein visualisation was inspected in regenerated putative transgenic lines. Leaf samples were prepared from putative transgenic and non-transgenic plants. The leaf explants were exposed to blue ultraviolet light at excitation bandpass of 400 nm with a fluorescence Olympus BX51 microscope (Olympus, Japan) equipped with narrowband filter of 460–490 nm. Images were captured using a digital camera (Olympus, Japan) attached to a computer.

### PCR characterization of the transgenic plants

Genomic DNA from putative transgenic and control plants was extracted using CTAB method^19^. The presence of a *GFP* and a *GFP-GUS* fragments were examined using polymerase chain reaction (PCR). The designed primers for *GFP* and *GFP-GUS* fragments were (F: 5′TCGTGACCACCCTGACCTAC3′, R: 5′ACCTTGATGCCGTTCTTCTGC3′) and (F: 5′AGGACGACGGCAACTACAAG3′, R: 5′TGACCCACACTTTGCCGTAA3′) respectively. These primer pairs were basically designed to amplify a 310 bp fragment of *GFP* and 711 bp of *GFP-GUS*. The PCR reaction mix in a final volume of 50 μL contained water, 50 ng template DNA, 5 pmol of each primer, 2.5 mM of each dNTPs, 2.5 mM MgCl_2_ and 0.5 U of *Taq* polymerase. Amplification reaction was defined as follows; denaturation at 94 °C for 5 min, 35 subsequent cycles of 94 °C for 30 s (denaturation), 58 °C for 30 s (annealing) and 72 °C for 45 s (polymerisation), and final extension at 72 °C for 8 min. The linear pXK2FS7 plasmid was used as the positive control. PCR products were finally visualised on 1.2% (w/v) agarose gel.

### Assessment of transgene expression

Total RNA was extracted from leaves of positive PCR transgenic and control plants (non-transgenic) using Mazzara and James method^20^. To avoid PCR amplification from genomic DNA, the RNA was treated with DNase I (DNAbiotech). First-strand cDNA was synthesized from total RNA using 2X HyperScript RT premix (GeneAll, South Korea) according to the manufacturer instructions. The cDNA was then used for PCR amplification of a 300 bp of *GFP* using specific primers (F: 5'TCGTGACCACCCTGACCTAC3', R: 5'ACCTTGATGCCGTTCTTCTGC3'). PCR products were finally visualised on 1.2% (w/v) agarose gel.

### In vitro verification of stable transformation

To evaluate transient and stable transformation, in vitro regeneration of T_0_ generation was performed. The stem's nodes of confirmed transgenic plants were isolated, and cultured on the MS medium containing PGRs and 60 mg l^−1^ kanamycin. Following regeneration, a number of regenerated shoots were assessed using observation of GFP or molecular assessment (PCR and RT-PCR).

### Data analysis

Treatments are assigned to a completely randomized design (CRD) with three replicates of ten explants. The data were analysed with SPSS 15.0 software package. Comparison of means was performed using Duncan's multiple range test (*p* ≤ 0.05).

## Results

### In vitro plant regeneration

**Figure 2:**
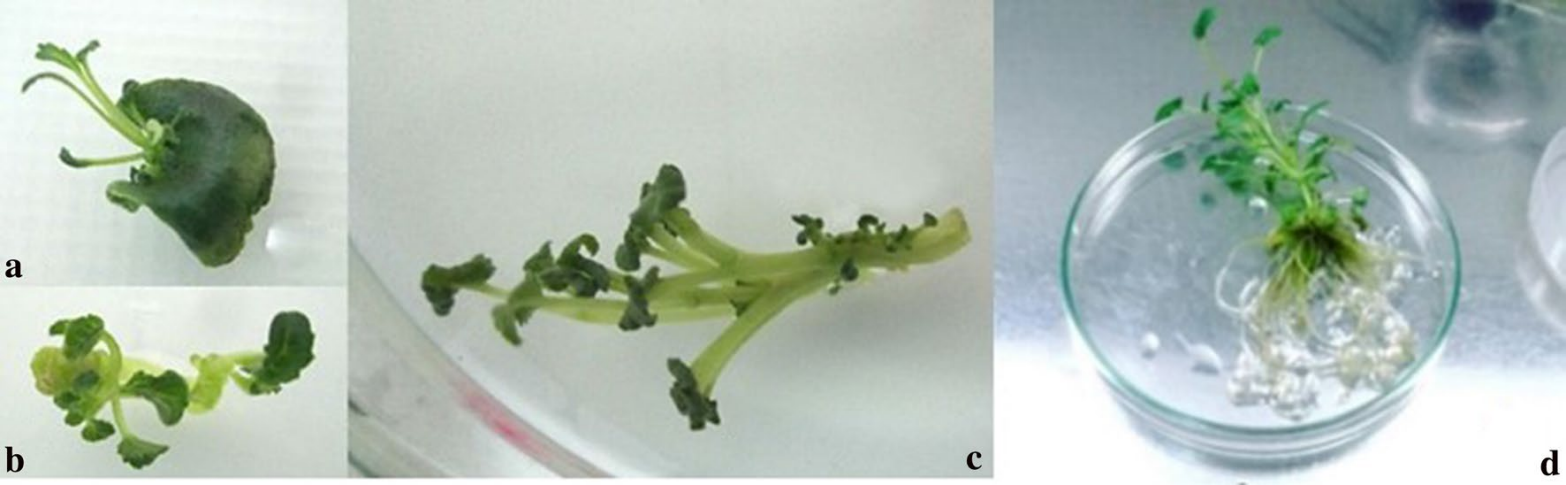
Response of explants on plant regeneration medium containing 1 mg l^−1^ BAP plus 0.05 mg l^−1^ NAA. (**a,b**) direct shoot formation after two weeks on cotyledonary node; (**c**) direct shoot regeneration from hypocotyl; (**d**) roots induced after two weeks on medium containing 0.1 mg l^−1^ NAA.

In regeneration experiment, cotyledonary node, cotyledon, hypocotyl and leaf explants were examined on 16 different PGR combinations (Table 1). Cotyledonary nodes were able to directly regenerate shoots in all PGR combinations (Fig. 2a) except control (0 concentration of PGR) or media without BAP. But, no shoots or calli were obtained on cotyledon and leaf explants in the presence of the 16 combinations, nonetheless hypocotyls produced a few shoots only on the medium supplemented with 2 mg l^−1^ BAP + 0.1 mg l^−1^ NAA (Fig. 2, b and c). Shoot regeneration frequency (Table 1) and the number of induced shoots per explant (Table 2) in cotyledonary nodes were significantly varied in different combinations of NAA/BAP. The highest regeneration rate (100%) and number of induced shoots (23 ± 3.60) were observed in combination of 1 mg l^−1^ BAP + 0.05 mg l^−1^ NAA. However, other combinations resulted 100% regeneration occurrence but the number of induced shoots was significantly lower than of 1 mg l^−1^ BAP + 0.05 mg l^−1^ NAA combination (Table 2). The lowest regeneration rate (59.66) was obtained from medium containing 2 mg l^−1^ BAP plus 0.05 mg l^−1^ NAA and the lowest number of induced shoots (3.33 ± 1.15%) was resulted in the medium containing 2 mg l^−1^ BAP (Table 1 and 2). Overall, different responses were observed in different explant types and better regeneration performance for cotyledonary node was obtained using 0.5 or 1 mg l^−1^ BAP in combination with some concentrations of NAA (Tables 1 and 2). Shoot regeneration from cotyledonary node and hypocotyl explants are presented in Fig. 2.

**Table 1:** Effect of different concentrations of BAP/ NAA on shoot regeneration frequency from cotyledonary nodes of *L*. *iberica*.

NAA (mg l^−1^)	BAP (mg l^−1^)			
	0	0.5	1	2
0	0.00 ± 0.00	86.33 ± 6.50^b^	68.33 ± 4.04^de^	59.66 ± 5.50^ef^
0.05	0.00 ± 0.00	84 ± 3.46^bc^	100 ± 0.00^a^	55.33 ± 4.04^f^
0.1	0.00 ± 0.00	73 ± 7.00^ cd^	100 ± 0.00^a^	73 ± 6.08^ cd^
0.2	0.00 ± 0.00	82 ± 10.14^bc^	75.33 ± 2.08^bcd^	100 ± 0.00^a^

Means with only the same letters are not significantly different at the 5% level based on Duncan’s multiple range test (*p* ≤ 0.05). Data shown represent means of regeneration frequency ± SD (*n* = 3).

**Table 2:** Number of induced shoots from cotyledonary node explants.

NAA (mg l^−1^)	BAP (mg l^−1^)			
	0	0.5	1	2
0	0.00 ± 0.00	15 ± 1.00^bc^	13.33 ± 1.52^ cd^	3.33 ± 1.15^ g^
0.05	0.00 ± 0.00	14.33 ± 0.57^bc^	23 ± 3.60^a^	6.33 ± 2.00^ef^
0.1	0.00 ± 0.00	11.66 ± 1.52^d^	16.33 ± 2.30^b^	7.33 ± 2.08^e^
0.2	0.00 ± 0.00	15 ± 1.00^bc^	14.33 ± 2.51^bc^	8.66 ± 2.51^e^

Means with only the same letters are not significantly different at the 5% level based on Duncan’s multiple range test (*p* ≤ 0.05). Data shown represent means of number of shoots ± SD (*n* = 3).

### Root induction

**Figure 3:**
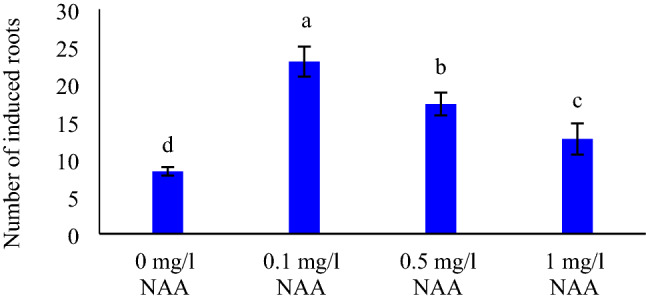
The effect of different concentrations of NAA on root induction. Means with only the same letters are not significantly different at the 5% level based on Duncan’s multiple range test (p ≤ 0.05). Data shown represent means of induced roots ± SD (n = 3).

Roots were initiated after nearby two weeks on in vitro-regenerated shoots cultured on MS medium with different concentrations of NAA (Fig. 2d). Maximum (23 ± 2.00) and minimum (8.33 ± 0.57) number of roots were induced in the medium supplemented with 0.1 mg l^−1^ NAA and 0 mg l^−1^ NAA, respectively. Furthermore, by increasing the concentration of NAA over 0.1 mg l^−1^, number of induced roots was reduced (*p* ≤ 0.05) (Fig. 3).

### Acclimatization of plantlets

**Figure 4:**
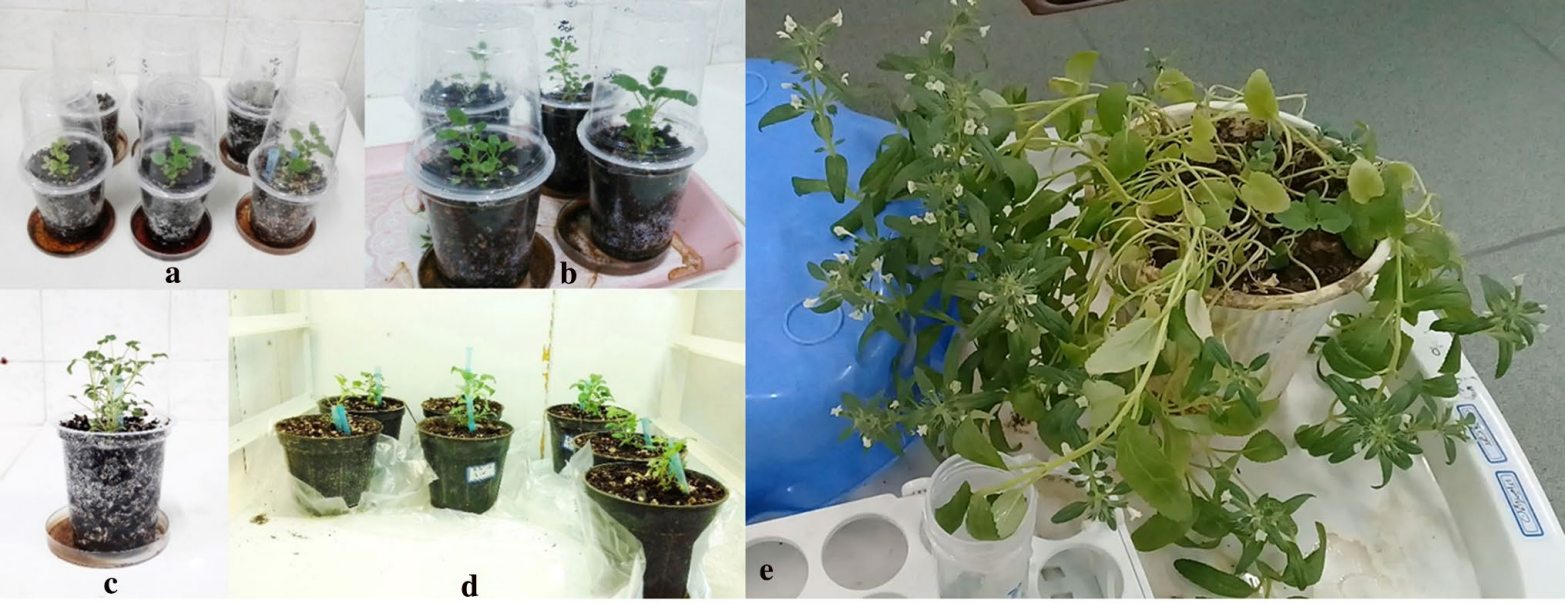
Acclimatization of regenerated plantlets. (**a**) The early stages of adaptation: transfer the plantlets to the pots containing 1:1 ratio of coco peat and perlite mix and covering them with transparent plastic shield; (**b**) making 2–3 holes in the shields to easy adapt plants; (**c**) removing of the plastic cover after 3–4 weeks; (**d**) transfer of plantlets to larger pots containing mixture of farm soil, compost, and sand at the late stage of adaptation; (**e**) a plant at flourishing stage.

The in vitro-regenerated shoots with well-developed root system were maintained in the acclimatization conditions for 4 weeks in the small plastic pots (Fig. 4a,b,c) contain coco peat and perlite mix (1:1) and presented the best results with 100% survival rates. The plantlets were next transferred to the greenhouse into the larger pots filled with 1: 1: 1 ratio of farm soil, compost and sand (Fig. 4d), compost and sand and they grew and developed normally and morphologically were identical to the mother plant. Overall, the regenerated plants were morphologically normal, phenotypically stable, fertile, reproductive and able to set viable seeds (Fig. 4e).

### Kanamycin tolerance assay

**Figure 5:**
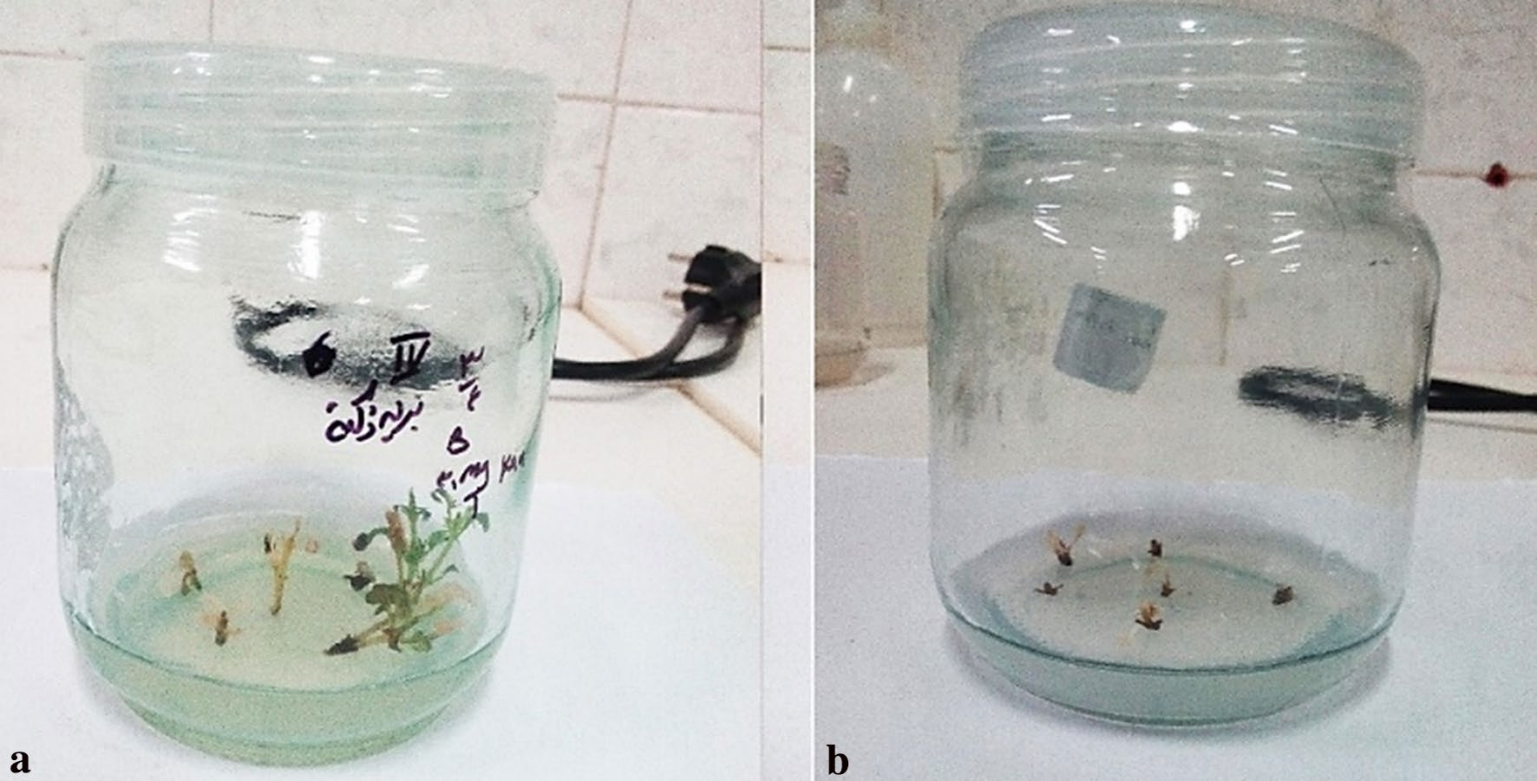
Explants on regeneration medium containing 30 mg l^−1^ and 60 mg l^−1^ kanamycin. (**a**) Explants on a medium containing 30 mg l^−1^ kanamycin after 3 weeks; (**b**) explants on a medium containing 60 mg l^−1^ kanamycin after 3 weeks.

In the control (0 concentration of kanamycin), all explants exhibited normal growth and were healthy and green. On medium containing 30 mg l^−1^ kanamycin, some explants stopped growing (Fig. 5a) and their tissues turned brown after 30 days but most of them survived (66.66 ± 0.57%). In all other applied levels of kanamycin (60, 90 and 120 mg l^−1^, the explants were in poor condition and after two weeks they showed gradual whitening and finally stopped growing (Fig. 5b). Therefore, the concentration of 60 mg l^−1^ was designated as the effective level of kanamycin for selection of putative transgenic plants.

### Plant transformation

**Figure 6:**
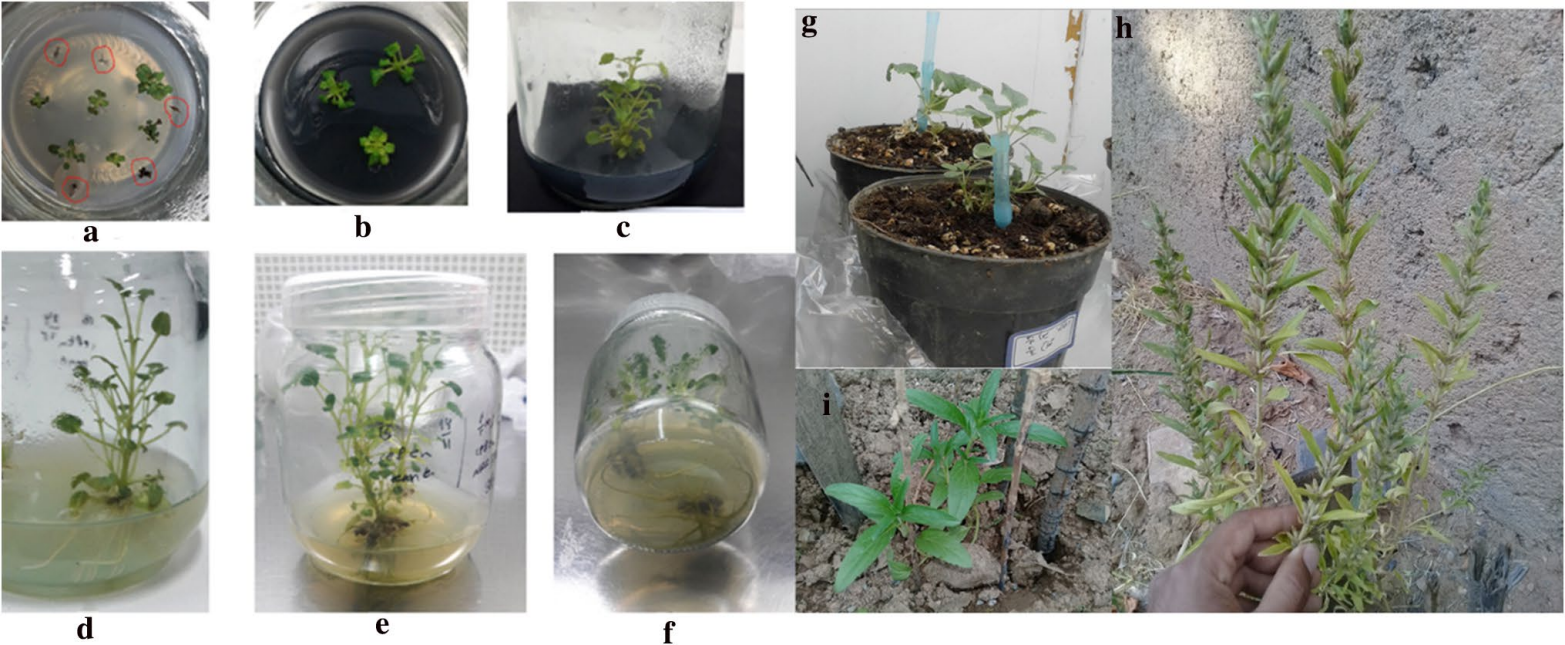
Regeneration of explants in selective medium containing 60 mg l^−1^ Kanamycin plus 400 mg l^−1^ cefotaxime after inoculation of explants with *Agrobacterium* cells. (**a**) Regeneration of putative transgenic plantlets (green plantlets) and non-transformed plantlets (Red circles) after two weeks of culture; (**b**) Sub-culturing of putative transgenic plantlets; (**c**) further development of plantlets in a new medium; (**d**,**e**,**f**) root initiation of regenerated shoot in MS medium containing 0.1 mg l^−1^ NAA and 60 mg l^−1^ kanamycin; (**g**) acclimatized plantlets; (**h**) mature plant producing seeds; (**i**) T_1_ transgenic progeny plants.

Using optimized regeneration system, explant transformation was performed and finally several shoots were successfully induced from cotyledonary nodes in regeneration medium (1 mg l^−1^ BAP + 0.05 mg l^−1^ NAA) containing 60 mg l^−1^ kanamycin (Fig. 6a,b). Four weeks after culture, shoots were entirely developed (Fig. 6c). The induced shoots were gently transferred into rooting medium (including 0.1 mg l^−1^ NAA) (Fig. 6d,e,f). Finally, the rooted plants were acclimatized and hardened before moving them to the greenhouse for further analysis (Fig. 6g). There were no obvious phenotype differences between normal wild type and transgenic plants. They were fertile, reproductive and able to set viable seeds (Fig. 6h).

### Effect of infection time on transformation efficiency

**Figure 7:**
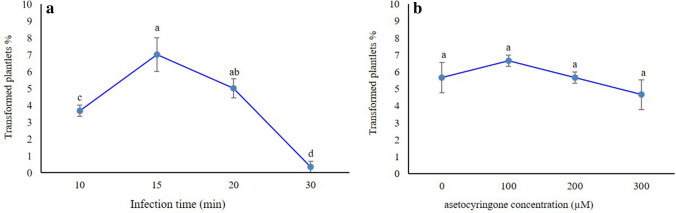
The effect of (**a**) different infection time and (**b**) concentrations of acetosyringone on gene transfer percentage. Means with only the same letters are not significantly different at the 5% level based on Duncan’s multiple range test (*p* ≤ 0.05). Data shown represent means of transformation frequency ± SD (*n* = 3).

Analysis of variance showed that the effect of exposure time (treatment) of explants to *Agrobacterium* was significant. It means that the different infection time resulted different transformation efficiency. Comparison of means using Duncan's multiple range test (p ≤ 0.05) revealed that the highest transformation efficiency (7 ± 2.51%) was occurred at infection time of 15 min (Fig. 7a). By increasing of the infection period in *Agrobacterium* suspension, the transformation efficiency rates were considerably reduced (Fig. 7a).

### Effect of acetosyringone on transformation efficiency

Maximum percentage of transformation (6.66 ± 0.33%) was achieved when acetosyringone was applied at 100 µM, however, presence or absence of acetosyringone did not significantly affect the transformation efficiency (*p* ≤ 0.05; Fig. 7b). Higher concentrations of acetosyringone (200 and 300 µM) caused overgrowth of agrobacteria and browning of explants, ultimately leading to death of some explants.

### Evaluation of transgenesis by GFP visualization

**Figure 8:**
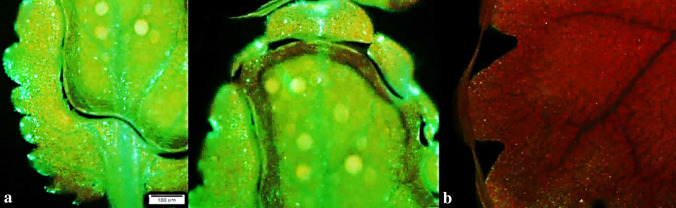
Green Fluorescence emission of green leaves from greenhouse-grown putative transgenic plants, (**a**) leaf sample from putative transgenic plant, (**b**) leaf segment of a wild-type plant (non-transgenic).

To confirm the transgene expression in regenerated plants after transformation, leaf samples of putative transgenic and non-transgenic plants (control) were investigated under fluorescence Olympus BX51 microscope (Olympus, Japan). All the collected sample leaves from different putative transgenic plants exhibited obviously green fluorescence signals emitted by all leaf parts under UV excitation (Fig. 8a), while no signal of green fluorescence was observed in the non-transgenic leaf samples and they appeared red (Fig. 8b).

### Confirmation of putative transgenic lines by PCR

**Figure 9:**
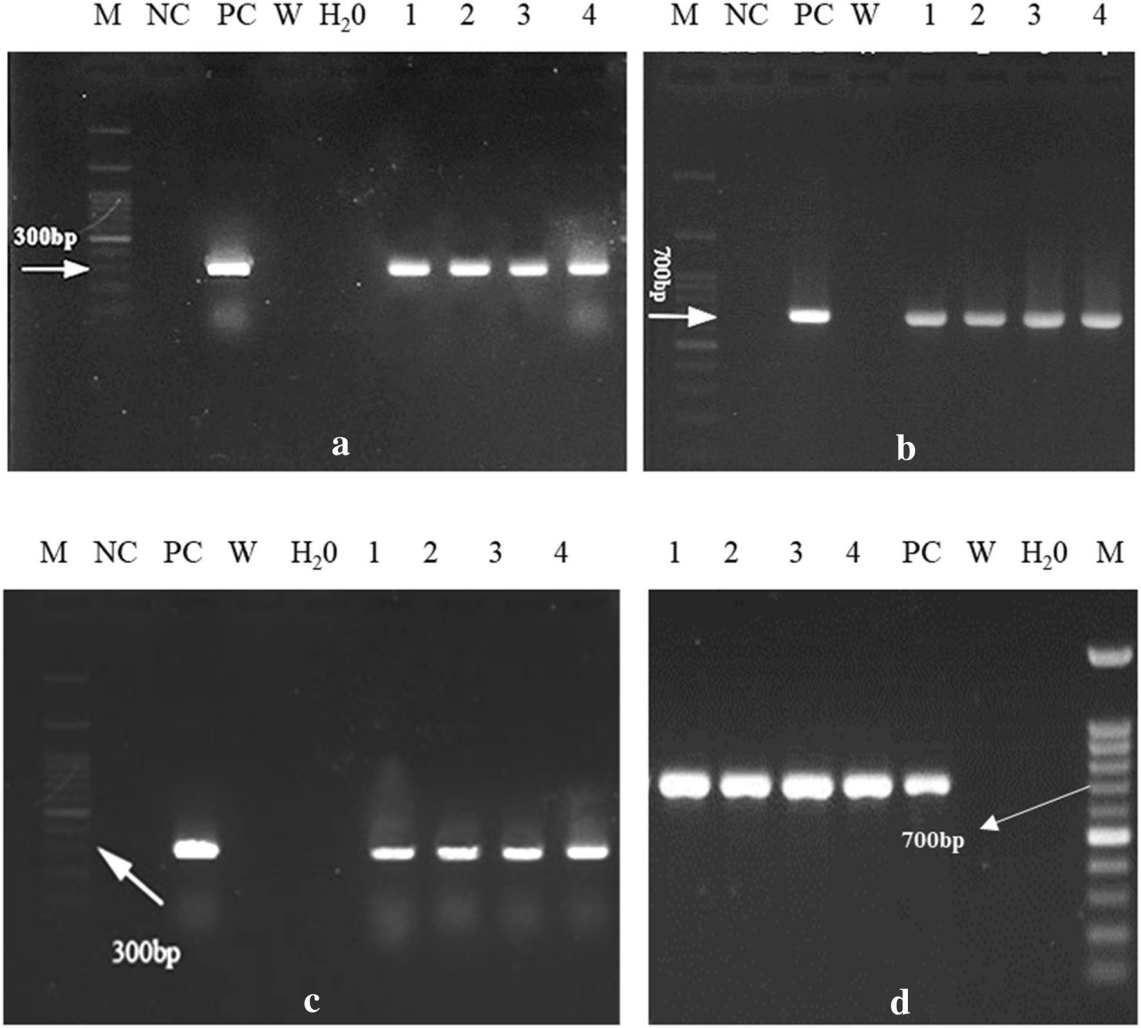
Gel electrophoresis of PCR on genomic DNA from the putative transgenic T_0_ plants using the (**a**) *GFP* and (**b**) *GFP-GUS* primers; (**c**) gel electrophoresis of PCR on cDNA from the putative transgenic T_0_ plants using the *GFP* primers (**d**) PCR on DNA from four T_1_ plants using the *GFP-GUS* primers. M) DNA Ladder 100 bp; NC) Negative Control; PC) Positive Control containing linear plasmid of binary vector; W) non-transgenic plants; lanes 1, 2, 3 and 4 are transgenic lines screened by GFP visualisation.

PCR reactions were carried out using primers designed specifically for the DNA region of interest. Agarose gel profile representing the PCR products showed exact amplicons of 310 bp for *GFP* (Fig. 9a) and 710 bp for *GFP-GUS* (Fig. 9b) located on the T-DNA. No band was detected on genomic DNA of non-transgenic plants. The results of PCR confirmed the successful transfer and integration of T-DNA in different plants. DNA was also subjected to PCR to detect *A. tumefaciens* DNA and thus residual agrobacteria and samples were found to be negative (data not shown).

### Analysis of transgene expression in transgenic plants

Transcript transgene expression was studied by RT-PCR. First, cDNA was synthesized from RNA samples of transgenic and non-transgenic plants which previously confirmed by PCR. RT-PCR was performed using specific primers to detect *GFP* transcript from transgenic plants. The RT-PCR product after agarose gel electrophoresis revealed an expected amplified fragment of 300 bp (Fig. 9c). While in non-transgenic plants no band was detected. This result confirmed successful integration and transcription of the genes on T-DNA in selected transgenic plants.

### Verification of stable transformation

The stem's nodes of confirmed T_0_ transgenic plants were sub-cultured onto the medium containing 60 mg l^−1^ kanamycin. Following the regeneration, a number of shoots were obtained. Leaf samples of the regenerated plants were evaluated under a fluorescence microscope. The GFP signals were effectively observed. In addition, the transgenic status of regenerated shoots was confirmed by PCR and RT-PCR and the targeted transgene fragments were detected. Additionally, PCR (Fig. 9d) was carried to detect a 711 bp *GFP-GUS* fragment on the genomic DNA of a few T_1_ plants (progeny plants) derived from primary transgenic plants (T_0_). T_1_ transgenic progeny plants did not show any considerable difference of morphological traits over the wild type plants (Fig. 6i).

## Discussion

Plant tissue culture method has been used as an important approach for secondary metabolites production, micropropagation and it can assist breeding for the genetic modification and improvement of medicinal plants. Therefore, establishment of an efficient tissue culture system with several applications could be a prerequisite for *L. iberica*. In the present study, for the first time, we achieved in vitro regeneration and *A*. *tumefaciens*-mediated transformation of *L. iberica*. Regeneration experiment revealed that the cotyledonary nodes exhibited the highest regenerative capacity. Other explants except hypocotyl failed to develop any callus or shoot and turned brown at last. These results support the importance of evaluating different explant types when developing a plant regeneration protocol. Nodal sections normally exhibit a high regeneration response. The cotyledonary node usually has many meristematic cells; therefore, this enables cotyledonary node to produce directly a large number of shoots. Several studies have demonstrated that cotyledonary node was the most appropriate explant for regeneration^21–23^ and it performed best for in vitro regeneration or *Agrobacterium*-mediated transformation in several plant species such as *Lupinus albus*^24^, *Passiflora caerulea L*.^25^, *Cicer arietinum L.*^26^, *Lallemantia iberica*^6^, *Glycine max*^27^ and *Stevia rebaudiana* Bertoni^28^.

The BAP in combination with NAA has been usually used for the shoot regeneration in various plant species^29–31^. BAP appears to be one of the most efficient cytokinins for shoot generation from nodal segments and the success of shoot induction using BAP is very high^32,33^. In *L. iberica* the BAP/NAA combination, at certain concentration, was successfully induced in vitro shoots from cotyledonary nodes. The optimal concentration of BAP/NAA combination for shoot regeneration was 1 mg l^−1^ BAP plus 0.05 mg l^−1^ NAA which led to induction of the highest number of shoots. In regeneration experiment of *L. iberica* the number of shoots were reduced by increasing the concentration of BAP to 2 mg^-l^, such results have also been reported in grain legumes^34,35^. Although the higher concentration of cytokinin can induce the signal of shoot formation, it may also inhibit the normal growth of shoots and can form vitrified tissues because of their toxicity as this has been reported previously in other plant species^36–39^. We found different performance of various explants and different PGR combinations in *L. iberica* regeneration. Previous studies in several plant species have also discovered that many factors such as genotype, the nature of explant, culture media type, combination and concentration of plant growth regulators can influence shoot regeneration^10,40,41^.

In root induction study, cluster roots were produced at all concentrations of NAA, but the maximum number of roots were induced in the medium supplemented with 0.1 mg l^−1^ NAA. On this NAA concentration vigorous roots developed in normal shape with branched and whitish colour. Furthermore, by increasing NAA concentration, the number of roots were significantly decreased. These results are consistent with study of Hsieh and colleagues^42^ which reported that the lowest concentration of NAA is appropriate to induce in vitro roots in *Arachis hypogaea*. Additionally, in *Siratia grosvenorii* the maximum roots observed in medium containing 0.1 mg l^−1^ NAA^43^. Indirect plant regeneration from callus is normally laborious and rely on different factors and additionally it might lead to somaclonal variation. Several researchers have declared that establishment of a tissue culture method for propagation and transformation from callus may result in genetic abnormalities^44–47^. Therefore, availability of a direct regeneration procedure is valuable for micropropagation and genetic transformation.

In *Agrobacterium-*mediated transformation experiment, two factors affecting gene transfer such as infection time and acetosyringone concentration were studied. The results showed that transformation efficiency of *L. iberica* was substantially affected by infection time, nonetheless the different concentrations of acetosyringone had no significant effect on transformation efficiency, although lower concentration of acetosyringone (100 $\mu M$) induced higher transformation efficiency (Fig. 7b). Phenolic compounds such as acetosyringone and hydroxy- acetosyringone, which attracts the agrobacteria to the wounded explants, also could induce the virulence genes, thus facilitating the transfer of the T-DNA region. Moreover, the transformation frequency might be negatively affected with the higher concentration of acetosyringone but the data was not statistically significant. Transformation efficiency was decreased at higher concentrations in other plant species such as *Anthurium andraeanum*^48^ and *Vanda* Kasem’s Delight^49^. Moreover, by increasing the time of explant inoculation, the number of obtained transgenic plants were intensely decreased, and no transgenic was obtained in the time of 30 min. Such effects of suitable time of inoculation on transformation efficiency was also reported in *Ocimum gratissimum*^39^.

Effect of kanamycin at four concentrations (0, 30, 60, 90 and 120 mg l^−1^) on regeneration revealed that the concentration of 60 mg/l^−1^ resulted in a 66.66% shoot induction frequency. Regeneration on this kanamycin concentration was effective and gene transformation was finally confirmed by PCR and RT-PCR. Similar to our result, in *Salvia miltiorrhiza*, kanamycin at concentration of 30 and 60 mg l^−1^ was used to screen the transformant plantlets and the highest regeneration efficiency was obtained in the medium containing 60 mg l^−1^^50^.

In addition to *nptII* selectable marker, which is frequently used to select transformed plants, pXK2FS7 vector contains the GFP reporter gene. One advantage of *GFP* is that its expression analysis does not involve a destructive assay and visualization of GFP expression is performed with fluorescent microscopy allowing tracking progress throughout different stages^51^. In this study the putative transformant plants were initially screened using GFP signals. The use of green fluorescent protein (GFP) marker gene can reduce the time required for selection of transgenics^52,53^. Fluorescent marker genes allow visual detection and monitoring of transgene expression in transformed tissues and manual selection of transgenic plants even without using antibiotic or herbicide selection markers^54,55^. Detection of GFP signals under fluorescence microscope provided successfully the initial screening of transgenic plants that were finally confirmed by PCR and RT-PCR.

## Conclusion

In this study, we established a preliminary in vitro procedure for direct regeneration of *L. iberica* using cotyledonary node and also a transformation system using *A*. *tumefaciens*. To our knowledge, this is the first report of *Agrobacterium*-mediated transformation of *L. iberica* which could provide feasible practical basis for genetic transformation and breeding of this valuable medicinal plant and it is expected to be of commercial value either by improvement of *L*. *iberica* seed oil or secondary metabolites. Also, genetic transformation provides a powerful tool to study of gene function and gene editing that will contribute to trait improvement, functional genomics and metabolite engineering.
